# Supplementary material for: Excitation of epsilon-near-zero resonance in ultra-thin indium tin oxide shell embedded nanostructured optical fiber
Source: Sci Rep. 2018 Feb 5;8:2342. doi: 10.1038/s41598-018-19633-2 (PMC5799369; doi:10.1038/s41598-018-19633-2)
Supplement: Supplementary file 1 — Supporting Information [file 41598_2018_19633_MOESM1_ESM.docx]

Supporting Information for

Excitation of epsilon-near-zero resonance in ultra-thin indium tin oxide shell embedded nanostructured optical fiber

*Khant Minn^1^, Aleksei Anopchenko^1^, Jingyi Yang^1^, and Ho Wai Howard Lee ^1,2^ ^†^*

^1^Department of Physics, Baylor University, Waco, TX 76798, United States

^2^The Institute for Quantum Science and Engineering, Texas A&M University, College Station, TX 77843, United States

†Corresponding author e-mail: [Howard_Lee@Baylor.edu](mailto:Howard_Lee@Baylor.edu)

**S1. ENZ wavelength of ITO**

The complex refractive index of ITO ($\boldsymbol{n=}\sqrt{\boldsymbol{\varepsilon}}$) is calculated from the Drude model permittivity function:

$$\boldsymbol{\varepsilon}\left( \boldsymbol{\omega} \right)\boldsymbol{=}\boldsymbol{\varepsilon}_{\boldsymbol{\infty}}\boldsymbol{-}\frac{{\boldsymbol{\omega}_{\boldsymbol{p}}}^{\boldsymbol{2}}}{\boldsymbol{\omega}^{\boldsymbol{2}}\boldsymbol{+i\omega}\boldsymbol{\Gamma}}$$

with the large frequency limit $\varepsilon_{\infty}=3.6$, plasma frequency $\omega_{p}=3.3722 \times{10}^{15} s^{-1}$, and damping coefficient $\Gamma=2.0263 \times{10}^{14}s^{-1}$. Since $Re\left( \varepsilon\right)={Re(n)}^{2}-{Im(n)}^{2}$, the vanishing real part of permittivity can be seen in figure S1 as the intersection of real and imaginary parts of refractive index curves. To the left of this ENZ wavelength, large $Re(n)$ and small $Im(n)$ implies that the optical property of ITO is dielectric-like, while to the right at large wavelengths, larger imaginary than the real part of index, or equivalently, the negative real part of permittivity, implies metal-like behavior.

**Fig. S1:** Complex refractive index and permittivity of ITO as a function of free space wavelength. At the crossover of real and imaginary parts of the index, the real part of permittivity vanishes.**S2. Higher order modes of ENZ fiber**

At low wavelengths, the ENZ nanobore fiber can support four modes: HE11, HE21, TM01, TE01. However, only the fundamental HE11 mode (linearly polarized) is supported by the fiber at large wavelengths since the three higher order modes cut off below 900nm. We use finite difference method to solve Maxwell’s equations on a cross-section of the ENZ fiber with the 10 nm thick ITO shell at the wavelength of 600 nm. Each of the three higher order modes is tracked over a wavelength range to calculate the modal loss, defined as $loss=-20{log}_{10}e^{-2\pi k/\lambda_{0}}$, where $k$ is the imaginary part of the effective index. Near the cut off wavelength of each mode, the optical field gets more and more spread out into the cladding. As a result, the loss vanishes as the cut off wavelength is approached. Figure S2 shows the modal losses of those modes as a function of wavelength.


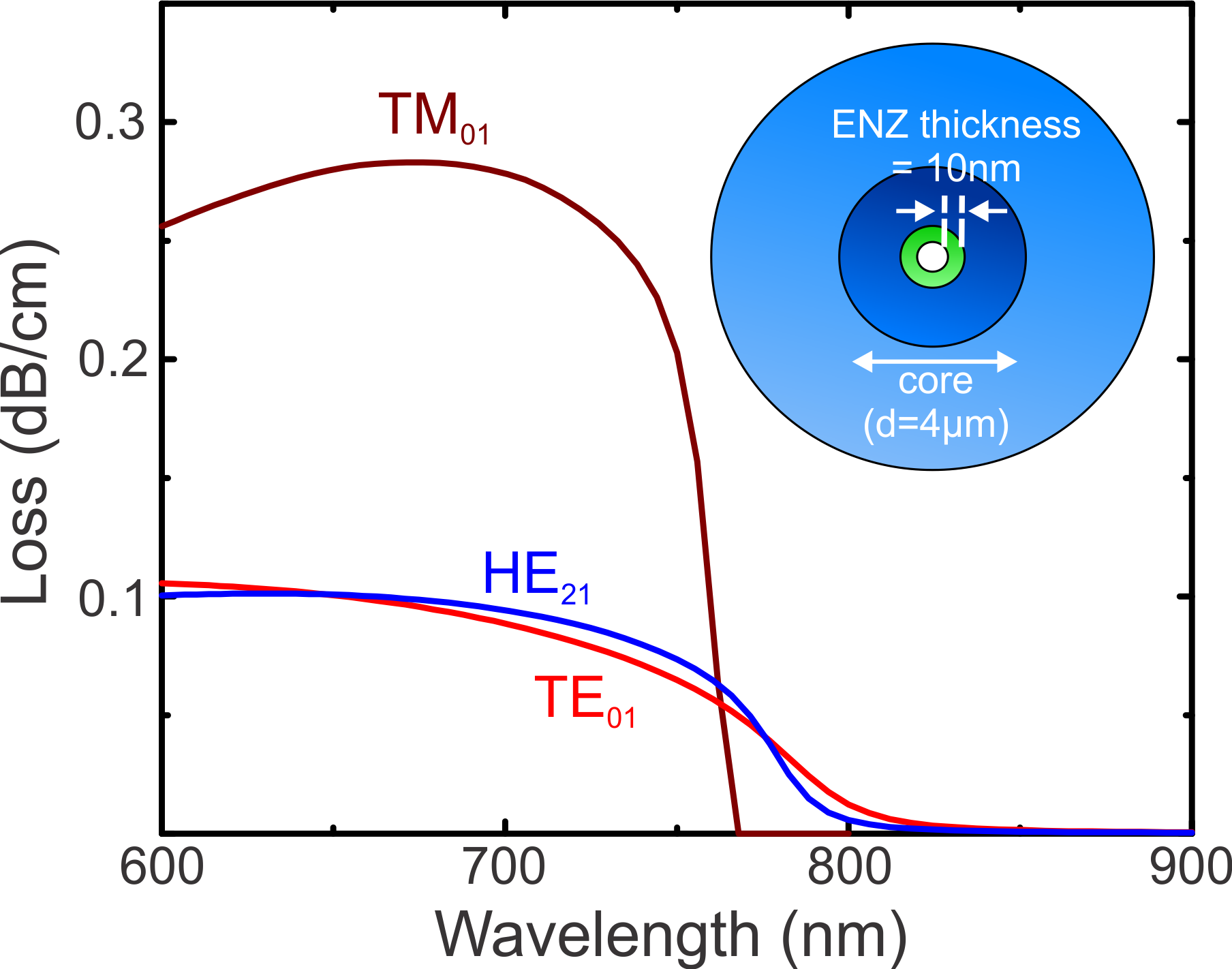


**Fig. S2:** Modal losses of ENZ fiber higher order modes. Loss vanishes as the mode approaches cutoff wavelength. The graph shows they cut off well below ENZ regime of ITO and thus, only the fundamental mode exhibits ENZ mode confinement.

**S3. Thin film ENZ mode in planar three layer geometry**

We investigate the three layer geometry shown in the insert of figure S3 (d) using transfer matrix method. The thin film ENZ mode is excited from the glass—the core material in the ENZ fiber—half space using Kretschmann configuration. The thickness of the ITO layer is 20 nm. The reflectance is calculated for varying incident angles and wavelengths for three exiting media: ethanol, acetonitrile and air. Light incident from glass onto ITO layer is perfectly absorbed at angles larger than the critical angle and at resonant wavelengths which corresponds to the ENZ mode. Contour plots of the reflectance as a function of incident angle and excitation wavelength is presented in figure S3 (a), (b) and (c) for ethanol, air and acetonitrile as exiting media respectively. The dashed white lines show the so-called light lines or the critical angle dispersion. The dotted white lines show the locus of the minimum reflectivity at a fixed incident angle or the ENZ mode dispersion. Electric field profile across the ITO layer and the exiting medium at the phase matching wavelength and angle is also calculated using transfer matrix simulations and plotted in figure S3 (d) for the three exiting media mentioned above. The field enhancement in the ITO layer is dependent on the exiting medium refractive index in agreement with the results reported in the main manuscript.


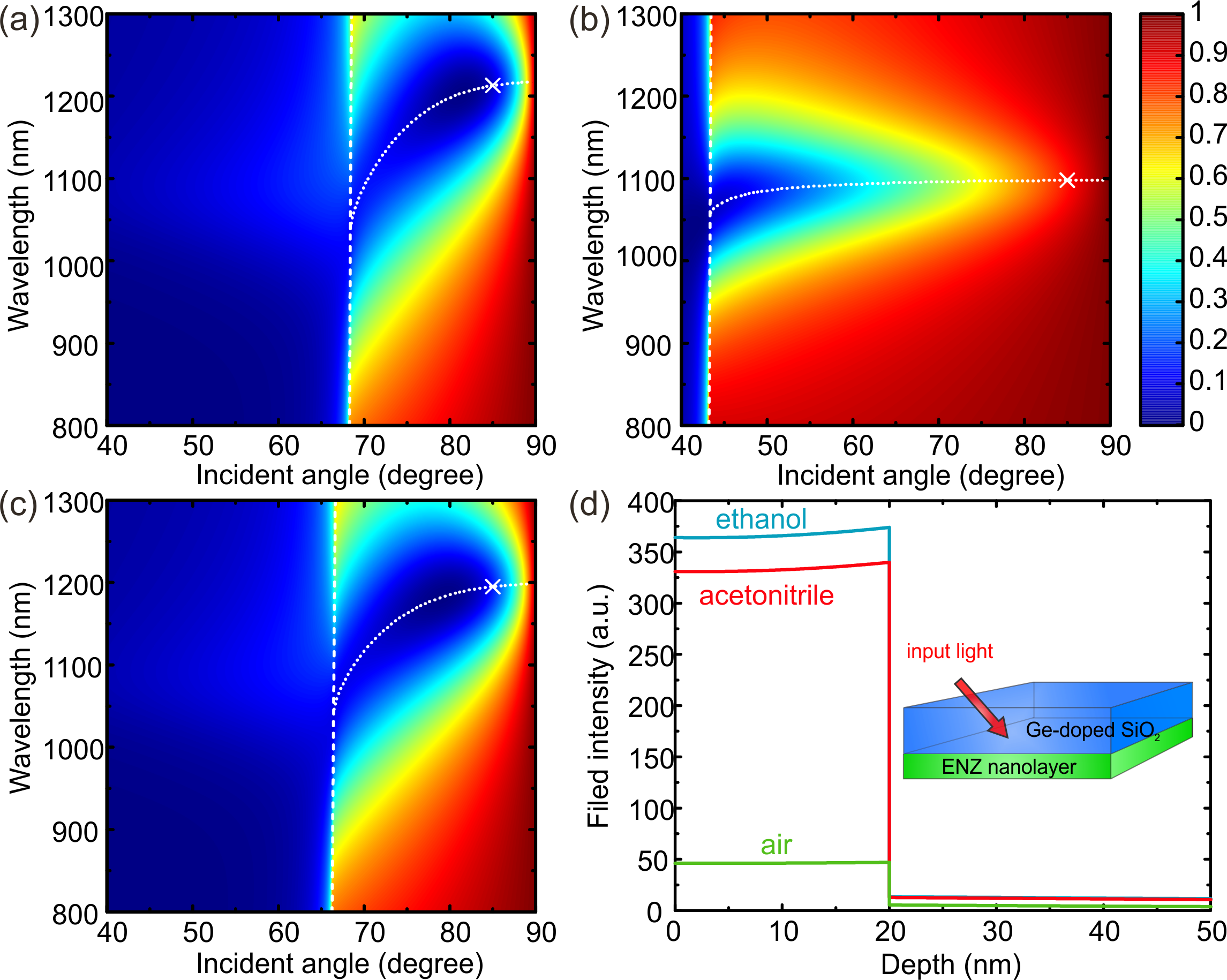


**Fig. S3:** Thin-film ENZ modes in three layer structure. Contour plot of reflectance as a function of wavelength and incident angle for (a) glass-ITO-ethanol, (b) glass-ITO-air, and (c) glass-ITO-acetonitrile structures. ITO layer thickness is 20 nm; the white dashed lines are the light lines/critical angles; the white dotted lines are the ENZ dispersion curves; and the white crosses are the phase-matching angles/wavelengths. (d) |E| inside the ITO layer for structures in (a), (b) and (c) calculated at the phase-matching wavelengths. Zero depth corresponds to the glass-ITO interface.
